# A computational assessment of the permeability and salt rejection of carbon nanotube membranes and their application to water desalination

**DOI:** 10.1098/rsta.2015.0020

**Published:** 2016-02-13

**Authors:** Michael Thomas, Ben Corry

**Affiliations:** 1 Department of Chemistry, La Trobe Institute for Molecular Science, La Trobe University, Melbourne, Victoria, Australia; 2 Research School of Biology, Australian National University, Canberra, Australian Capital Territory, Australia; 3 Life Sciences Computation Centre, Victorian Life Sciences Computation Initiative, Carlton, Victoria, Australia

**Keywords:** nanostructed membranes, molecular dynamics, seawater desalination, water filtration, water permeability

## Abstract

Membranes made from nanomaterials such as nanotubes and graphene have been suggested to have a range of applications in water filtration and desalination, but determining their suitability for these purposes requires an accurate assessment of the properties of these novel materials. In this study, we use molecular dynamics simulations to determine the permeability and salt rejection capabilities for membranes incorporating carbon nanotubes (CNTs) at a range of pore sizes, pressures and concentrations. We include the influence of osmotic gradients and concentration build up and simulate at realistic pressures to improve the reliability of estimated membrane transport properties. We find that salt rejection is highly dependent on the applied hydrostatic pressure, meaning high rejection can be achieved with wider tubes than previously thought; while membrane permeability depends on salt concentration. The ideal size of the CNTs for desalination applications yielding high permeability and high salt rejection is found to be around 1.1 nm diameter. While there are limited energy gains to be achieved in using ultra-permeable CNT membranes in desalination by reverse osmosis, such membranes may allow for smaller plants to be built as is required when size or weight must be minimized. There are diminishing returns in further increasing membrane permeability, so efforts should focus on the fabrication of membranes containing narrow or functionalized CNTs that yield the desired rejection or selection properties rather than trying to optimize pore densities.

## 1. Introduction

An increasing number of regions around the world are water stressed as climatic changes and population increases are reducing the reliability of fresh water sources [1]. It is estimated around 2 billion people lack access to safe drinking water [1] and in 2008, 3.4 million died from the use of unsafe water [2]. One way to address this problem is to increase the availability of potable water by treating salty or contaminated supplies to make them suitable for human consumption. This can be done with membrane filtration techniques such as microfiltration, ultrafiltration, nanofiltration and reverse osmosis that can be used to remove a wide range of solutes including large particles, bacteria, inorganic contaminants and salts. In all of these, water is forced through a selective barrier known as a semi-permeable membrane that blocks the passage of some of the solutes. What can be removed from the water and how much energy is required to do so depend upon the membrane characteristics. For example, the removal of small monovalent ions such as Na^+^ and Cl^−^ from water is particularly difficult, but is required if seawater or brackish groundwater are to be made suitable for drinking or agriculture. As a consequence, reverse osmosis desalination plants require considerable energy input in order to pressurize the feed water above the osmotic pressure difference so that it may flow through the membrane with a useful flux.

The most common membranes used to remove salt from water via reverse osmosis filtration are thin-film composites (TFCs). In these, a thin polyamide layer is placed on top of a more porous layer (usually made from polysulfone or polyethersulfone) which itself is bound to a fabric support. Salt rejection is achieved in the polyamide layer which contains narrow and tortuous, but continuous, pores which allow the flow of water, but impede the flow of salt ions. The degree of salt removal is intimately tied to the relative flow rates of water and ions, which are governed by the structure and composition of the membrane. Current TFC reverse osmosis membranes can achieve very high salt rejection (more than 99%) but have relatively low water permeability (approx. 1 l m^−2^ h^−1^ bar^−1^) [3]. In comparison, ultrafiltration membranes have poor salt rejection (less than 5%) but much greater water permeability (approx. 300 l m^−2^  h^−1^ bar^−1^) [3].

Materials incorporating carbon nanotubes (CNTs) and other nanomaterials have been touted as the next big leap in membrane technology. A number of experimental [4–13] and simulation [14–27] studies have noted that water can pass through CNTs and graphene pores much more rapidly than in conventional polymeric membrane materials. This flow is said to be near-frictionless, giving rise to an extraordinarily large slip length for this material. At the same time, reports of the salt rejection from CNT-based membranes suggest this can either be modest to very good depending on the system being investigated [11,15,17,21,23,28–30]. Although there are preliminary investigations of inorganic particulate removal in simplified pores [31,32] and nanofiltration membranes [33], how good CNT-based materials are at rejecting other contaminants has not yet been well studied. CNT-based membranes can differ widely in terms of the size of the nanotubes themselves, their orientation and density in the supporting material, the nature of the support itself and the presence of chemical functional groups on the nanotubes. Having an accurate assessment of the water permeability and rejection capabilities of CNT-based membranes with a range of different parameters is essential for understanding their potential uses in water filtration. However, manufacturing CNT membranes remains difficult, and so computational studies that can investigate a range of parameters are essential for directing the field. For example, such studies can help to determine the CNT types that yield the best water permeability while maintaining sufficient salt rejection to be useful in desalination technology.

Previous simulation studies have investigated the water fluxes and salt rejection in a variety of CNTs with differing pore size, functional groups, defects and lengths [11,17,18,21,23,24,34–36] Similar studies have also examined other nanotube types, such as silicon carbide and boron nitride [37] and pores in graphene sheets [27]. This study is conducted in a similar parameter space; however, there are two key differences from the previous work. Other studies have been conducted at a very high pressure, generally greater than 200 MPa (with one exception [38]), whereas membrane filtration and desalination plants operate at much lower pressures (less than or equal to 6 MPa). Pressures simulated in this study range from 5 to 400 MPa, allowing us to determine the permeability of the membrane, not just the water flux at a specific pressure. This means that we can examine how the properties of the membrane vary with driving force, and critically we can examine salt rejection and water flow at low pressures to see if the rapid flux and rejection arise under realistic operating conditions. The second difference is that here we include and maintain osmotic pressure gradients that have been poorly captured in previous simulation models. Improving our simulation conditions in this way enables concentrated ion layers on the membrane surface to be realistically modelled and their effects on water transport and ion rejection to be determined. These differences from previous work allow us to improve the estimate of the likely permeability and rejection properties of CNT membranes coming from simulation, explore parameters not yet studied computationally, and from this to assess the potential of nanotube membranes in desalination and filtration technologies.

In this study, we use molecular dynamics simulations to model a wide range of CNT-based filtration membranes, examining the influence of pore size, chemical functionality, pore density, pressure and salt concentration on permeability and salt rejection. In this case, we focus on relatively narrow pores (less than or equal to 1.6 nm in diameter) which may be suitable for desalination using reverse osmosis. From these data, we can make estimates of the potential benefits to power consumption and membrane area associated with employing these membranes in desalination facilities.

## 2. Methods

### (a) System set-up

**Figure 1.**
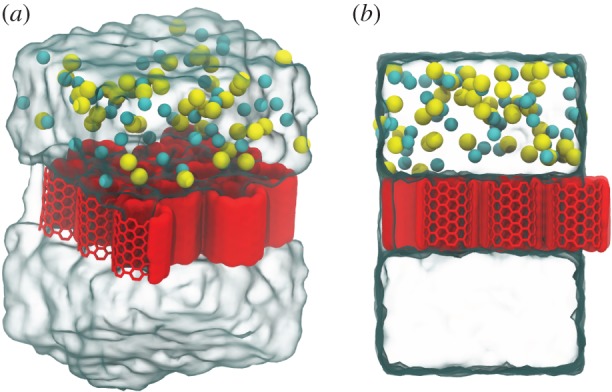
(*a*) Angled and (*b*) side view of the system of 4×3 array of CNTs used in this study. The nanotubes are represented in red, Na^+^ in yellow, Cl^−^ in cyan and the water as the transparentsurface. (Online version in colour.)

Each system is composed of a 4×3 array of hexagonally packed, armchair type nanotubes which is bounded by an upper salt water reservoir, and a lower fresh water reservoir, as depicted in figure 1. This system is periodic in all directions to produce an effective membrane sheet separated by layers of fresh and salty water. The water filled reservoirs are approximately 3 nm in height (approx. 8 Debye lengths for a 600 mM solution) ensuring that the electrostatic effects of ions in one CNT membrane are not felt by the next periodic copy. Membranes comprising armchair type CNTs of 1.4 nm in length were examined with results obtained for a range of CNT diameters as indicated in table 1. For simplicity, we examine CNTs of a single length as the effect of length has previously been examined by ourselves and others [25,39]. Using similar parameters to those utilized here, the water flow rate does not significantly change with small changes in CNT length [25], although the flow enhancement compared to continuum expectations does change with much longer CNT length even when the water in the CNT is not influenced by the simulation thermostat [39].

**Table 1.** The set of systems simulated in this study. Each system is simulated at six different pressures. The diameters of the nanotubes studied are reported as the distance between centres of opposing carbon atoms. (Note that the internal diameter of each CNT accounting for the size of the constituent atoms is approx. 0.34 nm less than the C–C diameter.)

CNT type	diameter (nm)	feed conc. (mM)	functional groups
(6,6)	0.66	600	—
(8,8)	1.09	300	—
(8,8)	1.09	600	—
(8,8)	1.09	1200	—
(8,8)	1.09	2400	—
(8,8)	1.09	600	2COO^−^
(10,10)	1.36	600	—
(10,10)	1.36	600	2COO^−^
(10,10)	1.36	600	COOH/COO^−^
(10,10)	1.36	600	2COOH
(12,12)	1.63	600	—
(12,12)	1.63	600	2COO^−^

**Figure 2.**
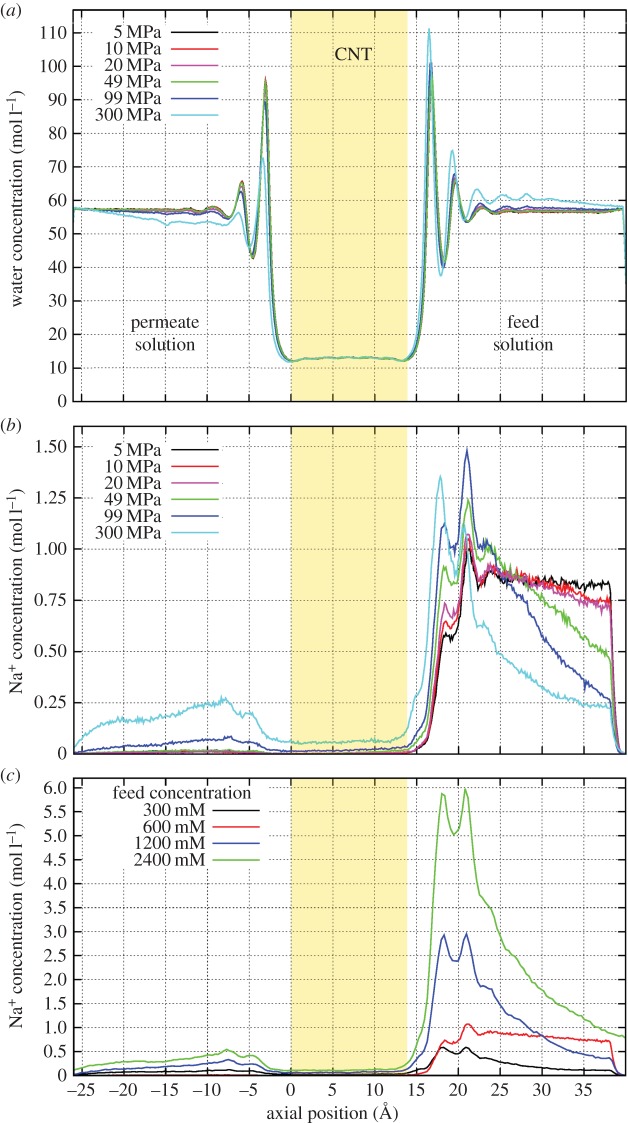
Concentration polarization of the pristine (8,8) system with 600 mM feed solution for various pressures for (*a*) water and (*b*) Na^+^. (*c*) The concentration polarization of Na^+^ for the same nanotube system under different NaCl feed concentrations at 200 MPa. The lower, fresh water reservoir is to the left, the upper, salt water reservoir isto the right and the CNT membrane composed of (8,8), 1.4 nm long nanotubes is the central region highlighted in orange. Similar results are obtained for Cl^−^ but are not shown here. (Online version in colour.)

Previous simulations by ourselves and others utilizing periodic boundaries have not been able to create different ion concentrations on the two sides of the membrane. This is a consequence of the two reservoirs being connected through the periodic boundary such that ions can move from one to the other without passing through the membrane. To overcome this limitation and allow for concentration differences to be established in the current study, a one-way boundary for ions is placed at the top of the salt water reservoir:
2.1
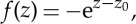
where *f*(*z*) is the force added to ions in the *z*-direction and *z*_0_ is the position at which the applied force begins to take effect. This prevents the back flow of ions from the salt water reservoir into the fresh water reservoir, which maintains a concentration gradient across the membrane (figure 2). This force is only applied to ions within 3 Å of the upper *z* periodic boundary.

Permeability and salt rejection are the metrics used to compare the performance of the membranes. A number of different parameters of the nanotubes and membranes are changed in order to establish their impact on these key measures. These parameters include the CNT pore radius, functionalization, hydrostatic pressure, as well as the salt concentration of the salt water reservoir and the packing density of the nanotubes in the membranes. A full list of the different simulation systems is given in table 1. Investigating the effect of each of these parameters have on the water flux and salt rejections allows us to construct a picture of how efficient, and how suitable, CNT-based membranes can be for desalination and filtration processes.

Functionalization of nanotubes often occurs during the manufacturing process: usually oxygen containing species such as carboxylate, carboxylic acid, hydroxyl and carbonyl groups are present at the ends of the tubes or in defects along the tube length [40]. It is prudent to this study to investigate the effect of terminal functional groups on permeability and salt rejection. In this study, the effects of −COO^−^, COOH and combinations thereof are studied. These functional groups are thought to have the largest effect on nanotube properties compared to other common oxygen-containing species due to their large electrostatic interactions with ions and water [6].

### (b) Simulation protocol

Parameters for the carbon atoms in the nanotubes are taken from the aromatic carbon ‘CA’ type in the CHARMM27 force field with a partial charge of 0.0 as done previously [17,21,23,25]. TIP3P [41] water molecules and ion parameters from Joung & Cheatham [42] are used. We have chosen to use the standard CHARMM parameters that are well validated for the use of small aromatic molecules in TIP3P water [43,44]. Alternative parameters have also been developed for graphitic carbon by, for example, validating against the contact angle of water on graphene [26]. As none of these validation metrics can provide a unique set of parameters, and given that we do not have experimental measurements of water transport in single well-characterized nanotubes to compare with, the choice of intermolecular potentials does add uncertainty into the transport predictions coming from our simulations.

Each carbon atom is restrained by a harmonic constraint of 0.1 kcal mol^−1^ Å^−1^ to prevent translation of the membrane when pressure is applied. The CNT/water/ion systems are minimized for 10 ps and then equilibrated for 1 ns under an NPT ensemble. After equilibration hydrostatic pressure is applied by way of a force on the oxygen atoms of the water molecules in the upper half of the upper reservoir, and the lower half of the lower reservoir, in line with the methods developed by Zhu & Schulten [45]. When applying hydrostatic pressure, the volume of the simulations is held constant (NVT ensemble) with the cell dimensions set at the final cell dimensions of the equilibration simulations. The actual hydrostatic pressure in each simulation is determined from Δ*P*=*nf*/*A*, where *n* is the average number of oxygen atoms to which these additional forces are applied during the simulation, *f* is the force applied to each oxygen atom and *A* is the cross-sectional area of the membrane. Simulations are conducted for 50–100 ns for each pressure, with lower pressures being simulated for longer times for statistical considerations. A plot of the water density versus *z*-position shown in figure 2*a* indicates that the density equilibrates in the half of the reservoir distant from the membrane, even at very high pressure. This indicates that the size of the periodic cells is sufficient for pressure equilibration far from the membrane.

All simulations are conducted using NAMD 2.9 [46] at a constant temperature of 298 K using a Lowe–Andersen thermostat, which has been demonstrated to provide reasonable dynamics for mass transport simulations [25], and 1 fs timesteps, a cut-off distance of 12 Å, a switching distance of 10 Åand a pair list distance of 13.5 Åwere used in each simulation. Particle mesh Ewald is used to calculate long-range electrostatic interactions. A Langevin piston is used to maintain a pressure of 0.1013 MPa during equilibration (NPT ensemble).

### (c) Analysis

Water flux, *F*, is determined by summing the number of water molecules undertaking a complete crossing of the membrane over a particular period of time. The permeability, *σ*, is given by
2.2
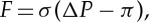
where Δ*P* is the pressure drop and *π* is the osmotic pressure. The flux and permeability are dependent on the number of pores per unit area, i.e. the pore density. For this, we assume two pore densities: an experimentally achieved density of CNTs, 2.5×10^11^ CNTs cm^−2^ [5], and the hypothetical maximum packing density, where CNTs are packed hexagonally side by side (i.e. there is no ‘matrix’ in which they are embedded). The first provides a reasonable estimate of permeabilities that we may expect in the foreseeable future, while the second provides theoretical limits to this technology.

Similarly, Na^+^ and Cl^−^ crossing events are counted to determine the percentage salt rejection, which is defined as
2.3
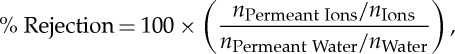
where *n*_Permeant Ions_ and *n*_Permeant Water_ are the number of water molecules and ions passing across the membrane during the simulation, *n*_Ions_ and *n*_Water_ are the total number of ions and water molecules in the simulation system. This definition of rejection has been used in a number of previous studies [6,17,23,37].

## 3. Results

### (a) Concentration polarization

Many simulation studies of permeation through nanotube membranes under hydrostatic pressure have not properly accounted for salt concentration gradients across the membrane. This is due to the difficulty in maintaining different ion concentrations in periodic simulation systems as ions can pass from the reservoir on one side of the membrane to that on the other without actually passing through the membrane. As a consequence, many existing studies including our own have not fully accounted for osmotic gradients and concentration polarization caused by the buildup of ions on one side of the membrane.

Accurately including concentration gradients in our simulations is essential to replicate the conditions found in desalination and filtration membranes. To implement this in molecular dynamics simulations, a one-way barrier for ions is introduced into each simulation system. This one-way barrier means that ions can only reach the fresh water reservoir by passing directly through the membrane. This allows much larger ion concentrations to be achieved in the upper reservoir than in the lower as demonstrated in figure 2.

Under all driving pressures, water is seen to form structured layers on the membrane surface, yielding a distinctive density pattern in figure 2*a*. These dynamic but structured layers have been noted previously [15,18,27] and are largely due to the entropic effects at the water/membrane interface and the desire of water molecules to maintain hydrogen bonded networks. Such concentrated layers have the potential to influence the passage of both water and ions into the membrane pores.

The influence of the water layers, along with the hydrostatic pressure and separation of the ions in the reservoirs is evident in plots of the ion concentration profile (figure 2*b*,*c*). At lower pressures (5–50 MPa) with a system wide average salt concentration of 300 mM and a membrane composed of (8,8) CNTS, the ion concentration in the fresh water reservoir is very small, while it varies between 0.7 and 1.1 mol l^−1^ in the salt water reservoir. The one way barrier included in our simulations has the desired effect of separating the salt water and fresh water reservoirs. The formation of a double layer composed of Na^+^ and Cl^−^ is apparent in figure 2*b*; the spacing between the peaks of the ions is 1.3 Å and corresponds to the spacing of the structured water layers. The double layer can be seen as the influence of the hydrostatic pressure driving the ions against the membrane surface. The concentration in the double layer can be significantly larger than the bulk ion concentration and acts to slow the movement of water molecules towards the membrane interface through increased electrostatic interactions as well as having the potential to alter the salt rejection. As the pressure increases, more ions are forced towards the membrane interface, increasing the concentration in the double layer. A second peak for each ion becomes more prominent near the membrane interface as the pressure increases. The larger pressure allows more ions to permeate through the membrane, increasing the concentration in the fresh water reservoir. The concentration of the double layer of ions that is driven against the membrane is also dependent on the feed reservoir concentration as shown in figure 2*c*. The double layer becomes more pronounced as the feed concentration increases, and the local concentration can reach many times the bulk value in the extreme cases.

The large concentrations of ions that build up on the membrane surface are the local instance of the effect of concentration polarization that is well known to limit the permeability of reverse osmosis membranes. Having ion concentrations reaching up to four times that of the feed solution will have a significant impact on the performance of these membranes. Not only does it create a large local osmotic gradient resisting water flow, as discussed below, it also reduces water flux due to the resistance felt by water passing through the concentrated layer.

### (b) Permeability

**Figure 3.**
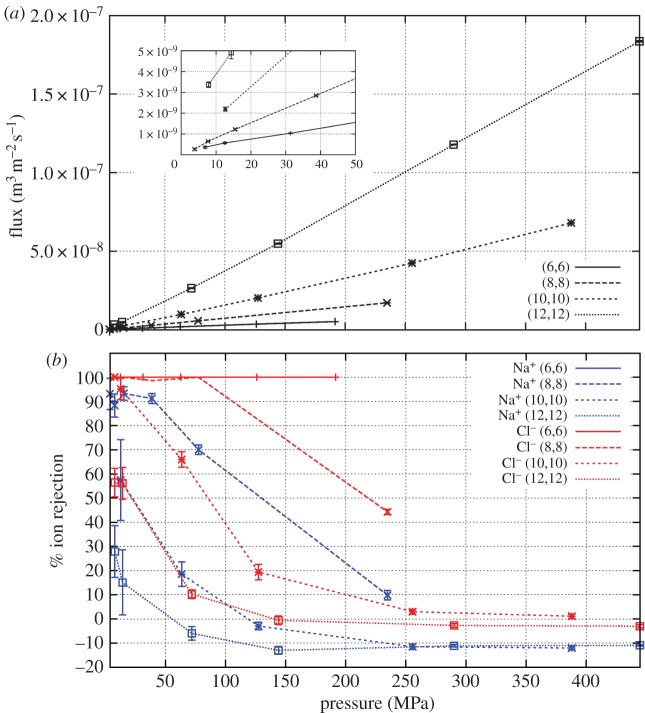
(*a*) The flux and (*b*) ion rejection response to hydrostatic pressure for various pristine tube types with 600 mM NaCl feed solution. Inset for (*a*) is zoomed in on 0–50 MPa. In (*b*), the Na^+^ (6,6) curve lies at 100%, underneath the Cl^−^ (6,6) line. (Online version in colour.)

The water flux and salt rejection of each membrane are determined for a range of pressures, a key difference between this study and others. The water flux increases linearly with pressure for each membrane simulated in this study; an example of this linearity is displayed for pristine nanotube membranes in figure 3*a*. This allows for the calculation (equation (2.2)) of the permeability of the membrane which is simply the gradient of the water flux with respect to the pressure.

**Figure 4.**
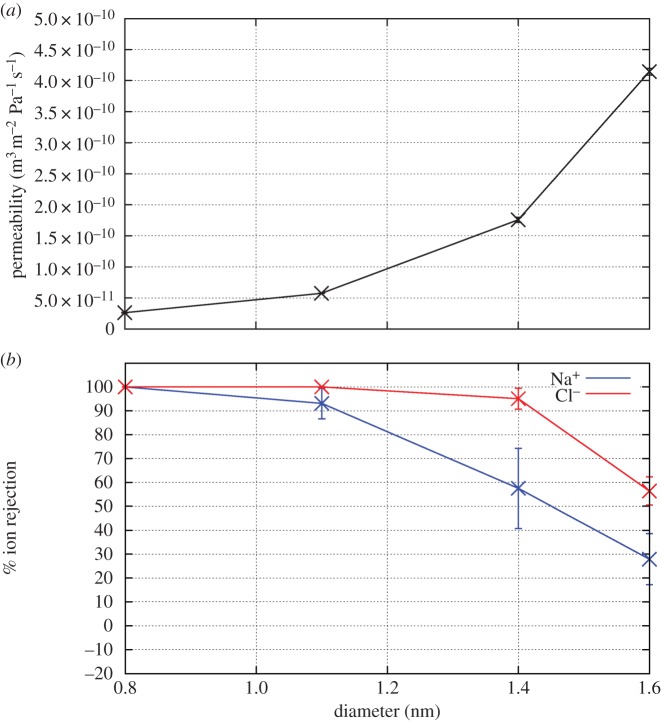
(*a*) The permeability and (*b*) ion rejection of pristine CNT membranes of various pore diameter in 600 mM NaCl feed solution. Ion rejection is determined at the lowest simulated pressure for each diameter. (Online version in colour.)

Generally speaking, permeabilities of maximally packed membranes are two to three orders of magnitude greater than those of membranes with experimentally achieved packing densities, which are themselves one to two orders of magnitude larger than those of the current best TFC membranes [3]. As expected, the permeability increases with nanotube diameter, as indicated in figure 4. Details about how various changes in conditions and nanotube structure influence permeabilities are described in the sections below.

The ability of water to flow rapidly through CNTs has been observed many times in simulation and experiment and is explained by the water sliding easily over the electrically and physically smooth nanotube walls [6,18,30]. Numerous computational studies have investigated whether water exhibits frictionless flow through CNTs. Several have concluded that flow is frictionless [15,17,24], while others have stated that it is not [47]. A prelude to the work presented here investigated this discrepancy and determined that much of this disparity was a result of the choice of simulation thermostat used in each investigation [25]. Some thermostats add additional ‘frictional’ forces to control the temperature of thermostated particles. This introduces non-physical frictional forces for water permeating nanotubes, causing membrane permeability to decrease with increasing nanotube length. Studies that did not use these thermostats demonstrated close to frictionless water flow. A more recent study of much longer nanotubes has shown that frictional effects do become apparent at much longer nanotube lengths, even when the water inside the CNTs is not influenced by the simulation thermostat [39]. The near frictionless flow of water through the nanotubes creates a plug flow in which the permeability is proportional to the cross-sectional area of the nanotube. In this case, the relative amount of water in contact with the nanotube walls (boundary layers) does not influence the flow rates as it would in conventional laminar flow.

### (c) Salt rejection

Most previous computational studies investigating CNTs as desalination or filtration membranes have been conducted under large hydrostatic pressure (more than 200 MPa) to allow sufficient permeation events to be witnessed to calculate statistically reliable fluxes [17,19–21,23,24]. On the basis of these high-pressure simulations, some of these studies concluded that only CNTs with small pore radii (typically (6,6) or smaller) would provide the required level of salt rejection required to desalinate seawater to drinkable standards (approx. 95%) [17,21]. In this study, simulations were conducted for each membrane over a range of pressures, from 5 MPa up to 400 MPa. This allows for results to be obtained at pressures comparable to the standard conditions in most reverse osmosis desalination plants and to determine how the flux and rejection change with hydrostatic pressure.

Figure 3*b* shows an example of how the salt rejection varies with pressure. At high pressures, the (8,8) tubes display modest ion rejection, while the (10,10) and (12,12) tubes show very little rejection. However, as the pressure decreases, the salt rejection in each case increases; the (8,8) membranes reject nearly all Cl^−^ and 90% of Na^+^, (10,10) membranes reject about 90% of Cl^−^ and 60% Na^+^ and the (12,12) membranes show a 50–80% Cl^−^ and 20–40% Na^+^ rejection. A similar response is seen in each membrane analysed in this study. The greater rejection of Cl^−^ than Na^+^ in prisitine CNTs has been described previously [21,23] and occurs due to a greater barrier for Cl^−^ entry. This larger barrier is due to the fact that more of the solvation shell must be removed from the larger Cl^−^ ion compared to the smaller Na^+^ ion in order to enter the pore [21].

The greater salt rejection at low pressures can be explained by considering the free energy profile of an ion and a water molecule travelling through the membrane. We have previously shown that the origin of salt rejection in narrow pristine nanotubes originates from the energetic cost of fully or partially dehydrating the ions as required for the solvated ions to enter the narrow pores [17,21,30,31]. Similar dehydration barriers exist for water moving from bulk into the narrow pores as there can be a loss or rearrangement of hydrogen bonds. The net salt rejection of these suitably narrow pores therefore comes from comparing the relative sizes of the energy barriers seen by ions and water and how this compares to the driving forces acting to ‘push’ the molecules into the nanotube. The energetic barrier of entry for both Na^+^ and Cl^−^ is significantly larger than that of water [17]. Large pressures provide enough energy so that these barriers are easily overcome; the difference in barriers between the ions and water is very small compared to the total amount of energy available to push these particles across the membrane. Only in the very narrow CNTs, where steric effects are important, does significant salt rejection remain at high pressures. In contrast, at small pressures, the difference in these barriers become significant. There is not enough driving force for the ions to surmount their barrier to entry most of the time, but as the barrier for water is smaller, it is still easily overcome. This phenomenon is different from the commonly observed behaviour at much lower pressures, where salt rejection increases with pressure as the driving force for water begins to overcome the natural diffusion of ions through the membrane along their osmotic gradient.

Preferential permeation of Na^+^ over Cl^−^ (or vice versa) is possible in our simulations without creating a charge buildup on the membrane. This is due to the periodic boundary conditions that allow for ions passing through the membrane to be recycled to the feed reservoir. In reality, differential permeation of cations and anions is unlikely, as the charge separation across the membrane acts to equalize the permeability of each ion type. As a consequence in a real (non-periodic) desalination system, charge separation would act to yield the same rejection value for both ion types, with the value being set predominantly by the ion that has the hardest time permeating the membrane.

### (d) Dependence on nanotube diameter

As shown in figure 4, membranes consisting of tubes with larger radii demonstrate larger permeabilities with the permeability roughly scaling according to the cross-sectional area of the nanotube. Larger pores allow for more ‘columns’ of hydrogen bonded water molecules to traverse. These columns have been detailed in a number of previous studies [17,48,49].

As the pore radius increases, the ion rejection decreases. As described above, the cause of ion rejection is the energy cost associated with the ion fully or partly dehydrating so it may enter a tube [17]. For wider nanotubes, fewer water molecules need to be removed for the ions to enter the pore, resulting in a smaller dehydration barrier making ion permeation more likely.

Combining the data on salt rejection versus both nanotube diameter and hydrostatic pressure allows us to assess the best pristine CNTs to use for desalination applications. That these nanotubes display greater salt rejection at low pressure than high pressure means that wider CNTs can be used than previously thought. Indeed, CNTs with diameter approx. 1.1–1.2 nm appear to provide the best compromise between permeability (6×10^−11^ m^3^ m^−2^ Pa^−1^ *s*^−1^= 0.0052 l m^−2^ Pa^−1^ d^−1^) and salt rejection (more than 95%). Current nanotube manufacturing processes make it extremely difficult to control the pore radius; indeed, a distribution of pore sizes normally results. However, with larger tubes displaying some salt rejection, the distribution need not be as tightly controlled as may have originally been thought. A mixture of tubes distributed around this ‘goldilocks’ diameter may still provide sufficient salt rejection for use in the desalination of seawater.

A notable outcome of our results is that significant ion rejection due to partial dehydration should arise for nanotubes up to 1.3 nm diameter. However, such rejection has not been seen in experimental studies. For example, Fornasiero saw some ion rejection in sub 2 nm CNTs, but this was attributed to electrostatic affects as it was concentration and pH dependent [29,50]. Ions have been seen to pass through even narrower tubes (less than 1.4 nm) under an electric field, suggesting that even in these narrow tubes high degrees of salt rejection are not seen [51]. In our simulations, salt rejection rapidly declines in wider pores, and it is possible that the lack of dehydration-based rejection in the experimental studies is simply a result of having too many wide pores. Because each wide pore carries much more current than each narrow pore, it only takes a small percentage of wide pores to remove rejection. While this can explain the lack of rejection in the sub 2 nm pores, it does not fully explain the lack of rejection in the sub 1.4 nm pores. Trace amount of very wide pores or membrane defects could further add to the loss of rejection, or defects in the tubes themselves could aid ion permeation due to increasing polarity of the pore walls. It is also possible that inaccuracies in the inter-molecular potential could lead to the discrepancy with experimental results. Perhaps even more critically, the non-polarizable classical model used here could overestimate salt rejection as it does not account for any stabilization of ions in the pore from electronic polarization of the CNTs.

### (e) Dependence on salt concentration

The majority of systems in this study were simulated with 600 mM NaCl in the feed (upper) reservoir to replicate typical seawater. However, during the desalination process, the concentration of the saline water increases as fresh water is driven through the membrane (as noted below, the salt concentration increases along the length of a typical cross flow system). Generally, desalination facilities will aim for close to 50% recovery (the proportion of the feed water converted to fresh water), meaning that the salt concentration at the end of the membrane is double that of the initial concentration. In our case, we run each simulation at a single salt concentration. However, if we assume an initial salt water concentration of 600 mM, then the simulations at 1200 mM represent the highest feed concentrations for a reverse osmosis process operating at 50% recovery. To investigate the effect of these larger salt concentrations on membrane performance, we model (8,8) CNT membrane systems using various salt concentrations. Concentrations below and above the typical operating range are also shown to better highlight the trends and allow extrapolation to other desalination situations such as the application to brackish water.

**Figure 5.**
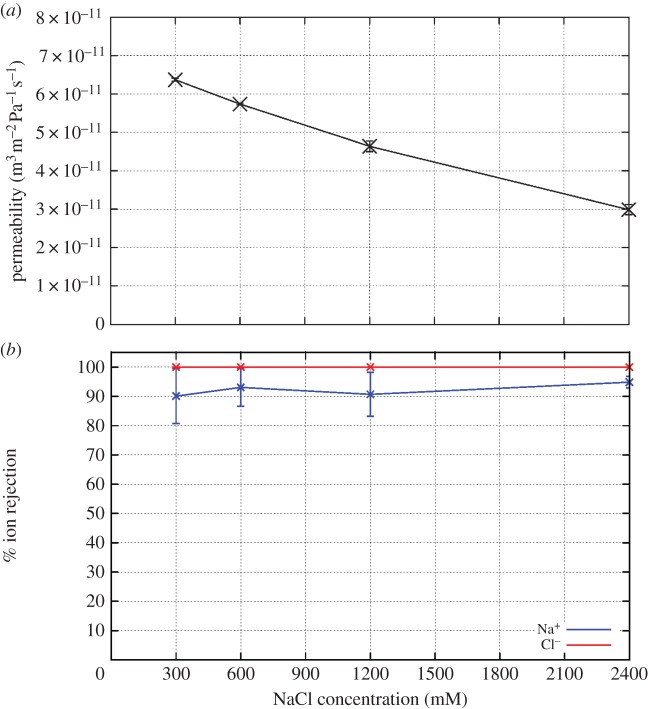
Influence of feed concentration on membrane permeability and salt rejection. (*a*) The water permeability of a membrane composed of (8,8) CNTs is plotted versus the average salt concentration in the feed reservoir. (*b*) The Na^+^ and Cl^−^ rejection is plotted versus salt concentration for an (8,8) CNT under a pressure of 5 MPa. (Online version in colour.)

Figure 5*a* shows that increasing the salt concentration in the upper reservoir decreases the permeability. This decrease in flow rates and permeability is due to two effects. Firstly, as the salt concentration increases, so too does the osmotic gradient and so the effective driving force for water flow declines and the flow rate drops. This yields a well-studied decline in flow rates at a given pressure, but will not influence the membrane permeability which is the slope of the flow rate with pressure. Secondly, the build up of salt ions near the interface of the membrane and water (figure 2) creates additional friction on the water flow. As water molecules must pass the ions in the double layer to enter the pores in the membrane, an increase in the number of ions means that water is subjected to increased electrostatic interactions before reaching the pore. This generates a frictional force on the flow, similar to what is seen when the pore entrances are functionalized with charged or polar moieties [23]. In addition, the larger salt concentration on the surface of the membrane at larger pressures means there is a greater local osmotic gradient to be overcome.

The ion rejection does not significantly change as the salt concentration in the feed reservoir changes (figure 5*b*). Because of the large barriers preventing ions from moving through the pores, they are better at preventing ions from passing than are TFC membranes that tend to show a decline in rejection at high salt concentrations. This behaviour of narrow CNT pores simplifies the design of desalination facilities as a constant salt rejection across the membrane can be assumed. Furthermore, an increase in osmotic pressure is not enough to alter the salt rejection.

### (f) Dependence on functionalization

**Figure 6.**
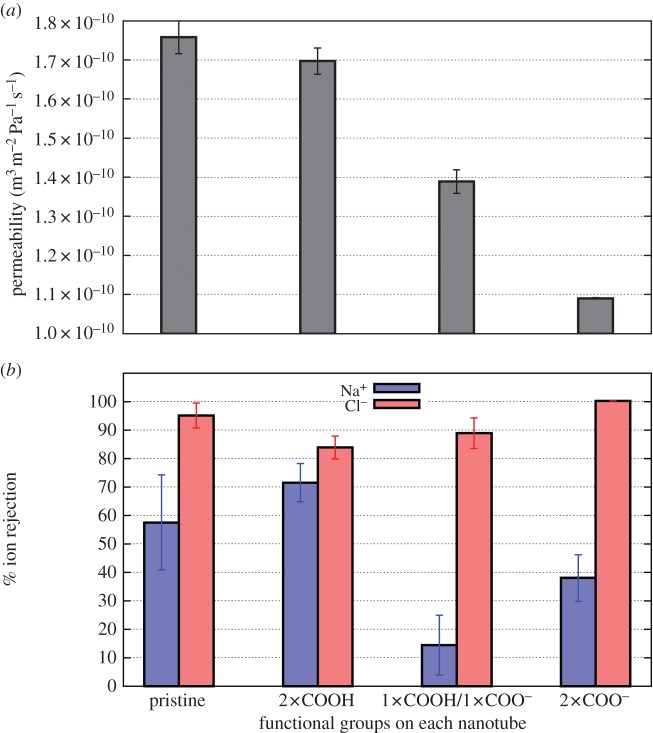
Comparison of (*a*) permeability and (*b*) salt rejection in pristine and functionalized nanotubes. Results are shown for a pristine (8,8) CNT, as well as for a similar CNT with two functional groups at each end of the pore as noted below each bar. (Online version in colour.)

The presence of functional groups at the end of the CNTs decreases the permeabilities of the membranes. As displayed in figure 6*a*, the neutral functional group, (8,8) COOH, exhibits only a small decrease in permeability from the pristine (8,8) nanotube, while the charged groups exhibit much larger decreases. The greater electrostatic interaction between the polar water and charged functional groups creates frictional forces that slow water transport [6,23].

There is only a small change in salt rejection of the (8,8) COOH functionalized nanotubes compared to the pristine (8,8). The largest changes occur in the membranes constructed of nanotubes with charged functional groups. There is an increasing in Cl^−^ rejection for both the mixed polar/charged and charged functionalized nanotubes. The introduction of a negative charge repels the negative Cl^−^ ion from the pore entrance, making it much less likely to permeate through the membrane. One would expect a similar phenomenon for positively charged functional groups and positive ions. When negatively charged functional groups are added to the pore (COOH/COO^−^ and 2×COO^−^), the Na^+^ rejection is not as good as for the pristine CNTs. This is to be expected as the negative charge attracts Na^+^ ions to the pore entrance aiding their entry to the pore and reducing the Na^+^ rejection. If the overall rejection is predominantly determined by the more strongly rejected ion as described above, then the best rejection would be achieved using charged functional groups in the pores. This accords with the good rejection that has been obtained in mixed matrix membranes containing zwitterionic functional groups at the pore mouths [11].

## 4. Discussion

Previous simulation studies of water transport and salt rejection in CNT membranes have examined conditions of very large hydrostatic pressures and poorly controlled ion concentrations. Here, we are able to overcome these limitations to provide the best estimates of the likely permeabilities and salt rejection of these materials, although we note there are still large uncertainties arising from the ill-defined inter-molecular potentials. As has been suggested previously [4–10,13,14–26], the flux through these membranes is very large, something which we can quantify here in terms of the membrane permeability. We are able to give an improved estimate from classical molecular dynamics simulations of the likely permeability of these narrow pores, with a membrane made from 1.1 nm diameter (8,8) CNT at a realistic pore density of 2.5×10^11^ cm^−2^ having a permeability of 5.7×10^−6^ m^3^ m^−2^ Pa^−1^ s^−1^, more than 16 times greater than the leading polymeric TFC reverse osmosis membrane. By simulating realistic conditions, we are also able to show that salt rejection improves as the hydrostatic pressure decreases. This means that wider CNTs can achieve better salt rejection than had previously been thought. The 1.1 nm diameter CNT is able to achieve better than 95% salt rejection in our simulations, which when coupled with its permeability places this diameter at the ideal size, or ‘goldilocks’ zone, obtaining the best compromise of rejection and permeability.

That CNT-based membranes could achieve such high permeability while maintaining salt rejection suggests they may be very beneficial for use in water filtration technologies. While TFC technology has been improving, the permeability of these membranes is only two times better than what it was 20 years ago [52]. A sudden change in permeability obtained through CNT-based membranes could have enormous implications. But, the impact of highly permeable membranes depends upon the specific application for which they are being used, the size of the potential gains in power consumption, the implications on plant and system design and the cost of manufacture. To begin to assess these issues, we look below at the energy requirements and design implications of CNT-based membranes for water desalination via reverse osmosis.

**Figure 7.**
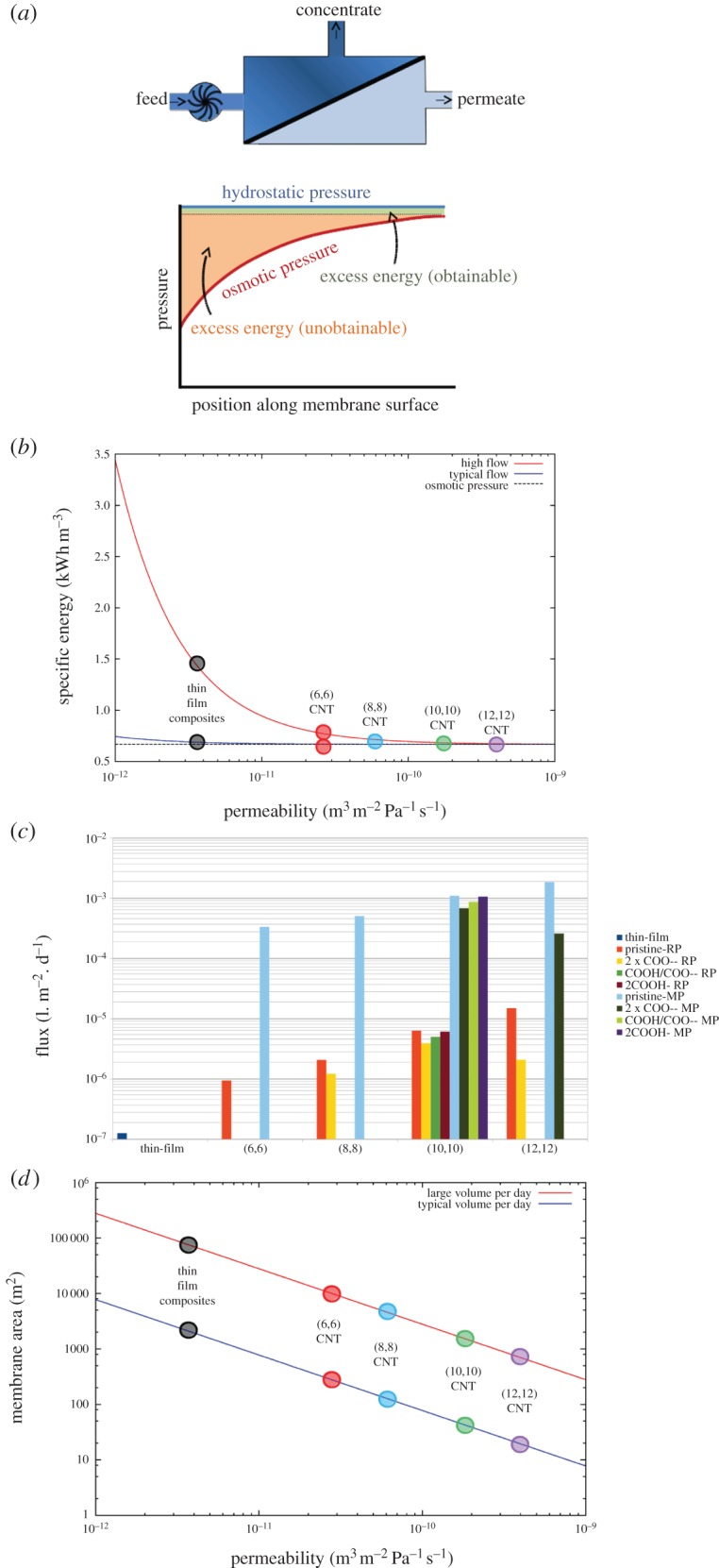
(*Caption opposite*.) The implications of CNT membranes for reverse osmosis. (*a*) Schematic of a cross flow reverse osmosis module and how the osmotic pressure changes along the membrane surface. Because the salt concentration increases along the surface, the flux decreases for a fixed hydrostatic pressure. The difference between the osmotic pressure and hydrostatic pressure represents the theoretical energy saving available,with only the area in green obtainable if the feed is pressurized once. (*b*) The energy required to achieve a given flux versus membrane permeability for high flow and low flow scenarios. Data points are shown for the best thin-film composite desalination membrane (Dow Filmtec SW30-HR380), and three membranes investigated in this study: unfunctionalized (8,8), (10,10) and (12,12) membranes at realistic pore densities. (*c*) The flux achievable for a given energy for pristine and functionalized nanotube membranes at achieved pore densities. (*d*) The membrane area required to produce a large volume (red line) and typical volume (blue line) of potable water per day in an idealized system with a 600 mM NaCl feed solution. The large volume assumes a flux of 1×10^−5^ m^3^ s^−1^, and 2.78×10^−7^ m^3^ s^−1^ for typical volume (for a 1 m^2^ membrane). (Online version in colour.)

The minimum hydrostatic pressure (and thus energy) required to operate a reverse osmosis desalination device is dictated by the salinity of the feed water and the desired recovery. In order to drive water across the membrane, the hydrostatic pressure must be greater than the osmotic pressure gradient which pulls water back from the fresh water side to the concentrated side. As the feed water becomes more concentrated, the osmotic pressure increases, and so the hydrostatic pressure requirement to keep producing fresh water also increases. To obtain a recovery of 50% for example, the final hydrostatic pressure must be greater than the osmotic pressure of a solution with twice the salt concentration of the feed. Modern desalination facilities operate in a cross flow mode as depicted in figure 7*a*, meaning that the salt concentration in the feed water increases along the membrane surface as fresh water is pushed through the membrane. The theoretical minimum energy required for this process is achieved when the applied hydrostatic pressure exactly matches the osmotic pressure [53] (although the flux is zero under these conditions so you would wait a long time to get a drink!). Commercial desalination devices operate at a single hydrostatic pressure rather than gradually increasing in pressure as the solution becomes more concentrated, due to the technical difficulties and added cost required to constantly repressurize the solution. The difference between the osmotic pressure and the hydrostatic pressure represents the added energy usage above the theoretical minimum as shown in figure 7*a*. When operating at a single pressure, the available energy saving is dictated by how much the pressure lies above the final osmotic pressure (green region in figure 7*a*) [53]. Seawater desalination facilities typically operate only marginally above the final osmotic pressure (approx. 5.83 MPa to achieve 45% recovery for 38 000 mg l^−1^ salinity [52]), thus there are only very small energy gains to be made by introducing more permeable membranes such as those described here when operating at a single hydrostatic pressure.

Rather than looking at the energy consumption in the complex conditions in a real desalination facility in which salt concentrations vary with position, to conceptually highlight the benefits that could be achieved using CNT membranes, we examine a simpler case with constant ion concentration. This analysis can be viewed as representing a single point in space along a cross flow membrane system (e.g. 600 mM NaCl represents the start of the membrane module for typical seawater desalination, while 1200 mM represents the end). At a given concentration *c*, the pressure Δ*P* required to achieve water flux *f* (volume of water passing through the membrane per unit area of membrane per unit time) can be determined by rearranging equation (2.2) [54]:
4.1
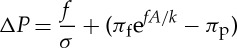
in which *σ* is the membrane permeability, *A* is the membrane area, *k* is the mass transfer coefficient, and *π*_f_ and *π*_p_ are the osmotic pressure in the feed and permeate reservoirs, respectively. The e^ *fA*/*k*^ relates to the concentration polarization factor due to the increased salt concentration on the membrane surface.

Assuming an idealized process (100% pumping efficiency, constant osmotic pressure across the length of the membrane, no other losses and no concentration polarization), the energy required to produce 1 m^3^ of water—the specific energy (SE)—is simply:
4.2
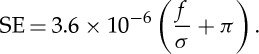


Figure 7*b* shows how much energy must be expended to achieve a particular flux of desalinated water for a range of membrane permeabilities when ignoring concentration polarization. As the membrane permeability increases, the amount of pressure over and above the osmotic pressure required to maintain a given flux decreases. Consequently, less energy is required to maintain a given flux. Thus, if achieving a large flux of fresh water for a given membrane area is the major factor dictating device design, then ultra-permeable membranes such as those described here could reduce the energy requirement. For the large flux case presented in figure 7*b* for example, the energy reduction can be as much as 50%, although frictional losses and concentration polarization cause additional problems when operating at high flux. In reality, the flux per unit area for a typical desalination plant is small, but the membrane area is large to produce significant amounts of potable water per day. In these cases, as can be seen by the lower line in figure 7*b* or in figure 7*c*, the energy savings are modest, highlighting the limited energy gains that can be achieved using ultra-permeable membranes when operating under standard conditions. An excellent analysis by Cohen-Tanugi *et al*. for the full cross-flow system with variable salt concentrations suggests that a 10% decrease in operating pressure (translating to a 15% energy saving) can be achievable for the reverse osmosis process using ultra-permeable membranes compared to operating parameters suggested by Dow Water (salinity 42 000 ppm, recovery 42% and operating pressure 7 MPa) [54]. However, much less improvement is available if we take as a reference the typical reference conditions suggested by Busch & Micklos (salinity 38 000 mg l^−1^, recovery 45%, operating pressure 5.83 MPa) [52]. In this case, the current operating pressure is only 1% above the outlet osmotic pressure, meaning there is only a 1% pressure saving that can be achieved. Cohen-Tanugi *et al*. point out, however, that there are significantly greater energy savings to be found using ultra-permeable membranes in brine desalination as operating pressures are generally far above the osmotic pressure of these feed solutions [54].

The amount of energy required to achieve a desired flux asymptotes to the osmotic pressure for very large values of permeabilities. A consequence of this is that there are diminishing energy savings obtained by further increasing the membrane permeability, even for the high flux conditions. The energy saving in moving from a polymeric TFC membrane (permeability approx. 0.4×10^−12^ m^3^ m^−2^ Pa^−1^ s^−1^) to one constructed from 1.1 nm CNTs (permeability approx. 6×10^−12^ m^3^ m^−2^ Pa^−1^ s^−1^) is much greater than that gained from changing from narrow CNTs to wider ones (e.g. 1.6 nm CNT, permeability approx. 40×10^−12^ m^3^ m^−2^ Pa^−1^ s^−1^). As the narrow CNTs have much better salt rejection than wider CNTs, efforts for making CNT membranes for desalination should focus on making the narrow CNTs for membranes, rather than trying to use wider tubes to increase the membrane permeability. Membrane permeability is also directly related to the pore density in the membrane. Given that the currently constructed CNT membranes already have very high permeabilities [5], there is probably little to be gained in terms of energy savings per volume of fresh water by focusing efforts on improving CNT packing densities. In figure 7*b*, we show data points for CNT membranes with currently obtained packing densities; data points for theoretical maximum packing densities are off to the right of the graph. There is little energy change (vertical shift), when improving the packing density with any of the CNTs studied. This can explain why including a small density of CNTs in a mixed matrix membrane can create a large increase in water flow [11,12]. However, for a given energy input, much larger fluxes can be obtained with more permeable membranes. There is about one to two orders of magnitude improvement for the nanotube membranes with obtained packing densities, and two to three orders of magnitude improvement for the maximally packed tubes in line with the increased permeabilities shown in figure 4.

Although the inclusion of chemical functional groups slows water flow as seen in figure 6*a* and shown previously [6], this does not have a major impact on the energy consumption to achieve a given flux. Figure 7*c* shows energy consumption for pristine nanotube-based membranes; decreases in permeability due to functional groups shift these data points slightly to the left in the asymptotic region. This results in very little difference in energy consumption between pristine and functionalized nanotubes.

While there are only small energy savings available from introducing highly permeable CNT-based reverse osmosis membranes into the desalination process, there is a major reduction possible in terms of the membrane area required to obtain a given amount of fresh water per day. Although CNT-based membranes could be expected to be more expensive than TFC membranes when they are first developed, because many fewer membrane elements are required, there may be savings in capital costs if the manufacture of ultra-permeable membranes can be made less expensive. Cohen-Tanugi *et al.* calculate that even a threefold improvement in permeability would allow for a 44% reduction in the number of membrane elements required to generate the same quantity of fresh water per day. In figure 7*d*, we show how the membrane area can be reduced while maintaining the existing conditions of reverse osmosis membranes with increased permeability. There is an inverse relationship between these parameters, which again highlights the diminishing returns with increasing permeability. Perhaps the most likely benefit of ultra-permeable CNT membranes in reverse osmosis would be the reduced footprint of the desalination facility. Places in which space is at a premium would benefit tremendously from the use of reverse osmosis membranes with improved water permeability. This could be particularly important on ships, oil rigs, space ships, islands, built-up zones or in mobile desalination facilities.

## 5. Conclusion

In this paper, we have presented a systematic simulation study of factors affecting the permeation and salt rejection of CNT-based filtration membranes which we hope can guide the future development of these materials. Importantly, we are able to show that salt rejection changes with hydrostatic pressure, meaning that better rejection can be achieved at realistic operating pressures. While the membrane permeability decreases at higher salt concentrations, salt rejection does not. Ultra-permeable membranes, such as those discussed here, do not have the capability to dramatically reduce the energy requirement for reverse osmosis desalination as current plants operate with pressures only a few per cent above the theoretical minimum for a single stage device. However, CNT-based membranes do have the potential to dramatically lower the required membrane area, something which could be beneficial when space is limited or for mobile units. As more potable water can be produced per unit area by CNT-based desalination membranes, these next generation membranes make an attractive alternative for water desalination or recycling on ships, oil rigs or space stations. The high permeability of these membranes compared to TFCs could also allow for them to be used in different ways, such as to obtain higher water flux and would show improved energy savings at high recovery. It is important that future work examine the performance of CNT membranes with respect to fouling and chloride resistance, both of which are factors that have large implications on their application; examine their use in ultrafiltration; and discuss whether they can be used to remove other water contaminants that are challenging to separate with polymeric membranes.
